# Privacy-friendly evaluation of patient data with secure multiparty computation in a European pilot study

**DOI:** 10.1038/s41746-024-01293-4

**Published:** 2024-10-14

**Authors:** Hendrik Ballhausen, Stefanie Corradini, Claus Belka, Dan Bogdanov, Luca Boldrini, Francesco Bono, Christian Goelz, Guillaume Landry, Giulia Panza, Katia Parodi, Riivo Talviste, Huong Elena Tran, Maria Antonietta Gambacorta, Sebastian Marschner

**Affiliations:** 1 Ludwig-Maximilians-Universität München, Munich, Germany; 2 Department of Radiation Oncology, LMU University Hospital, LMU Munich, Munich, Germany; 3 German Cancer Consortium (DKTK), Partner Site Munich, Munich, Germany; 4 Bavarian Cancer Research Center (BZKF), Munich, Germany; 5 Information Security Research Institute, Cybernetica AS, Tartu, Estonia; 6 Fondazione Policlinico Universitario “Agostino Gemelli” IRCCS, Rome, Italy; 7 Department of Medicine I, LMU University Hospital, LMU Munich, Munich, Germany

**Keywords:** Health care, Medical research, Metastasis

## Abstract

In multicentric studies, data sharing between institutions might negatively impact patient privacy or data security. An alternative is federated analysis by secure multiparty computation. This pilot study demonstrates an architecture and implementation addressing both technical challenges and legal difficulties in the particularly demanding setting of clinical research on cancer patients within the strict European regulation on patient privacy and data protection: 24 patients from LMU University Hospital in Munich, Germany, and 24 patients from Policlinico Universitario Fondazione Agostino Gemelli, Rome, Italy, were treated for adrenal gland metastasis with typically 40 Gy in 3 or 5 fractions of online-adaptive radiotherapy guided by real-time MR. High local control (21% complete remission, 27% partial remission, 40% stable disease) and low toxicity (73% reporting no toxicity) were observed. Median overall survival was 19 months. Federated analysis was found to improve clinical science through privacy-friendly evaluation of patient data in the European health data space.

## Introduction

Data has been heralded the “new oil” that fuels the digital economy. Society and science need data to make informed decisions, and such information becomes more reliable when it is built from many independent data sources. This is particularly true in medical research and personalized medicine. Rare diseases research requires collection of cases few and far between to reach meaningful case numbers. Personalized medicine requires tapping into diverse datasets scattered across health providers for each individual patient. E-health and digital epidemiology require federation of potentially millions of personal and remote IoT devices. In clinical research, the multicenter trial remains the gold standard for high-quality research, requiring data sharing or federation across partner sites^1^.

On the other hand, there is a lot of friction in sharing data openly. Patients are sensitized about sharing their data and are demanding an active say in their usage. Institutions and regulators are increasingly concerned about the privacy and legality of data sharing. Data protection and data security are ubiquitous, and informational self-determination has practically become a basic human right. Legislation such as General Data Protection Regulation (GDPR)^2^ or Health Insurance Portability and Accountability Act (HIPAA) form a tight corset for healthcare providers and research institutions to operate in. The solution to this conundrum will probably consist of both legal, societal, communicative, processual, and technological tools.

In particular, privacy-preserving technologies^3^ such as Secure Multiparty Computation (SMPC)^4^, Fully Homomorphic Encryption (FHE)^5,6^, or Differential Privacy (DP)^7,8^ promise to reconcile the need for data with the right to privacy by providing new mechanisms for collaboration and evaluation of data without the need to openly share such data. These technologies are often referred to as Privacy-Preserving Computation (PPC) in terms of classical statistical analysis or as Federated Analysis (FA) in terms of machine learning. As a new paradigm, PPC and FA promise to offer some beneficial perks: federation of heterogenous data sources across multiple organizations, avoiding of some of the legal and regulatory restrictions of data sharing, enabling of dynamic consent and dynamic retraction (right to forget), built-in mechanisms for data aggregation and metadata evaluation, and in general more democratic data flows (Open Data, Open Science, Citizen Science).

Some privacy-preserving computation technologies have been proposed or are already in active use in university medicine and clinical research^9–19^. For example, the German Medical Informatics Initiative (MII) is a joint effort of all German medical faculties to improve data sharing and data usage among their sites. Their task force for federated analysis has generated recommendations for data federation and is rolling out DataSHIELD^20,21^ to their partner sites. It is a semi decentralized system in which many data providers connect to a central Trusted Third Party (TTP). Researchers must then send their request to the central TTP which accesses the federated datasets and only returns the aggregate result. This system is very useful for example for feasibility analysis, where a researcher only needs summary statistics over multiple partner sites. Because DataSHIELD is already installed at many of MII’s partner sites, MII is generally recommending the use of DataSHIELD for PPC and has begun rolling out infrastructure to sites. Other tools were originally developed by MII researchers: The Trusted Server^22^ is a trusted computing environment, and EasySMPC^23^ is a no-code tool that aims to use existing infrastructure by communicating via email.

Our own group, within the German Consortium for Translational Cancer Research, had performed the first secure multiparty computation with real oncology patient data in Germany in 2019^24^. The radiation oncology departments of LMU Munich and Charité Berlin contributed *N* = 96 patients each. The Kaplan Meier estimator was implemented within the FRESCO framework with SPDZ protocol by an expert cryptographer from TU Munich. While the experiment was a success and allowed the analysis of confounding factors in the survival of patients suffering from glioblastoma, our team encountered problems that significantly restricted the long-term and reliable use of this promising technology. These included a complex tech stack, demanding implementation, complicated technology management, no prior standards for the data protection process, and a brain drain of our expert to the German Federal Office for Information Security immediately after the project.

The Federated Secure Computing (FSC) project was born consequently. The goal was to enable research institutions, government agencies, small and medium enterprises to deploy PPC solutions in a simpler fashion. During 2019 and 2020 we developed an easy-to-use propaedeutic architecture that offloads the complex and demanding cryptography to the server and separates the concerns of cryptography and business logic by providing a simple API^25^. In this way, existing powerful PPC solutions can be deployed behind this abstraction and easily be used by domain experts on the client side. The project was developed into a Free and Open Source (FOSS) initiative and has been supported and financed by Stifterverband, the German Donors’ Association, since 2021^26^. The express goal is to provide blueprints and visible show cases to address the full project chain from problem definition to technical solution, to regulatory implementation, to adoption by actual actors in the German and European data space.

In this paper, we present the first Secure Multiparty Computation with patient data across national borders within the European regulatory data space. Our lighthouse experiment provides:

- The use case of MR guided stereotactic body radiation therapy, a novel treatment modality warranting diligent quality control and regional cooperation to establish best practice.
- A rare tumor entity, metastases of the adrenal gland, requiring the pooling of data collected by multiple treatment centers to reach sufficient and significant statistics.
- The introduction of Sharemind MPC^27^ to university medicine, a commercial, feature-complete and industry-grade solution by the leading experts on secure multiparty computation.
- The use of Federated Secure Computing in a typical propaedeutic setting with several academic non-expert data providers and an academic non-expert researcher.
- The end-to-end documentation of the GDPR-compliant technical and administrative data protection measures that enabled regulators to green light this pilot experiment within the institutional, national and European regulatory framework on health data protection.

To our knowledge, this research constitutes the first ever use of true secure multiparty computation in the setting of an international clinical study.

We hope that this initiative might be useful as a blueprint for other groups to successfully implement and deploy Privacy-Preserving Computation and Federated Analysis to European healthcare data.

## Results

### Project timeline

The secure multiparty computation project started with an earlier pilot experiment in 2019 between LMU University Hospital and Charité^24^. In the follow-up of this proof-of-principle, the Federated Secure Computing Project by LMU Munich was launched. The team took part in a large initiative by Stifterverband and received coaching and funding through the years 2020 to 2024^25,26^.

The clinical study started in 2019 when the initial study protocol was written. The study protocol was amended and the cooperation agreement was signed in 2020 to allow for joint evaluation of data.

In November of 2022 the clinical study was proposed as a use case for the Federated Secure Computing project. From this point to the first calculation on live patient data the project duration was 14 months, including a number of preparatory steps. See Table 1.

**Table 1:** Project Timeline

Year	Milestone
2019	Earlier pilot experiment with secure multiparty computation in Germany
2020 to 2024	Federated Secure Computing initiative funded by Stifterverband
2019	Study protocol written
2020-05-13	Initial ethics vote
2020-07-06	Cooperation agreement signed
2020-11-30	Ethics vote amended to include cooperation agreement
2020 to 2023	Clinical data collection
2022-11-24	Clinical study proposed as use case for Federated Secure Computing project
2023-04-21	Munich data format shared
2023-04-26	Rome data format shared
2023-06-11	Data Use and Access Committee involved
2023-06-11	Data Protection Officers involved
2023-07-31	Recommendations by Data Use and Access Committee
2023-09-04	GDPR compliant documentation requested by Data Protection Officers
2023-09-21	Sharemind MPC Customer Agreement signed
2023-10-12	GDPR compliant documentation complete (98 pages)
2023-10-16	Permission granted by Data Protection Officers
2023-12-01	Sharemind MPC compute cluster functional
2023-12-18	Federated Secure Computing interface implemented
2023-12-18	System test with pseudo data complete
2023-12-21	Munich patient data goes live on the compute cluster
2024-01-25	Rome patient data goes live on the compute cluster
2024-01-25	First secure multiparty computation on patient data from both partner sites

Note that most of these steps would have been necessary also for conventional data sharing. The same legal pretext and similar data protection measures would have been needed. The clinical study and the data collection would have taken the same time. From the moment the data was available from both sides, the first calculations were done on the same day. The evaluation did not take longer because of the additional security. We did take some extra documentation steps during the overall project, though, to pave a way for future use of secure multiparty computation in clinical medicine.

### Federation

Patient data from both Munich and from Rome was successfully transmitted to the secure multiparty computation network. The upload time was about one second in both cases.

Both datasets each contained 24 rows (patients) and columns (data items). The data items included 20 categorial classifiers (e.g., sex, ECOG status) and 24 metrical floating-point variables (e.g., age, prescription dose, follow up time in months). When converted to CSV data, each dataset was about 15 kilobytes in size.

Exact timings are available for the Munich dataset. Of the total of 942 milliseconds, 69 milliseconds were spent on the client-side (reading the data from Microsoft Excel workbook, converting to CSV format). Another 457 milliseconds were spent on networking for four API calls (requesting a connection, requesting a microservice, uploading the data, and requesting the result). Another 154 milliseconds were spent server-side on the middleware (processing the API calls and feeding the data to the Sharemind MPC backend). Finally, 262 milliseconds or 28% of total computing time were spent on the backend (running Sharemind importer, distributing secret shares across servers). See Table 2.

**Table 2:** Computing time to upload data and distribute secret shares

Federated Secure Computing (client-side)	69 ms	7%
Networking (client-server and server-server)	457 ms	49%
Federated Secure Computing (server-side)	154 ms	16%
Middleware subtotal	680 ms	72%
Secure Multiparty Computation (backend server-side)	262 ms	28%
Total	942 ms	100%

HTTP request body payloads were 49 bytes for the connection POST request (71 bytes answer), no payload for the microservice GET request (71 bytes answer), 15,103 bytes for the upload PATCH request (71 bytes answer), and no payload for the result GET request (2662 bytes answer).

Similar to upload times, computing times were also generally below one second, see Table 3.

**Table 3:** Computing times for some Rmind statistical functions

function	on data from one partner site	on data from both partner sites
freq (frequency table)	0.31 s	0.38 s
mean	0.60 s	0.82 s
median	0.68 s	0.85 s
quantile (multiple quantiles)	0.70 s	0.96 s

### Clinical results

#### Patient characteristic

A total of 48 patients with adrenal gland metastasis were included from both centers. See Table 4 for detailed patient and therapy characteristics. Median age at radiotherapy initiation was 66 years (range: 38–91). The patients were predominantly male (60%) vs female (40%), without significant difference (*p* = 0.56). Main location of primary diagnosis was lung cancer with 63% followed by 9% with skin cancer. The Eastern Cooperative Oncology Group (ECOG) performance status was 0-1 in 40 patients (83%) and ECOG 2 in 8 patients (17%). 24 patients (50%) presented with metastasis in the left adrenal gland, 18 (38%) with metastasis on the right side and 6 patients (13%) presented with bilateral disease. Adrenal metastasis were metachronous, synchronous and oligoprogressive in 23 (48%), 13 (27%) and 8 (17%) patients respectively. In terms of metastatic pattern, 19% presented with solitary metastasis, 54% with oligometastatic disease and 25% with metastatic disease.

**Table 4:** Patient characteristic

		*n* (%)
Number of patients	Total	48
	Male	29 (60%)
	Female	19 (40%)
Age at treatment start	Median (range)	66 (38–91)
ECOG	0	18 (38%)
	1	22 (46%)
	2	8 (17%)
Laterality	Left	24 (50%)
	Right	18 (38%)
	Bilateral	6 (13%)
Timing of metastases	Metachronous	23 (48%)
	Oligoprogressive	13 (27%)
	Synchronous	8 (17%)
	Other	4 (8%)
Metastatic pattern	Solitary	9 (19%)
	Oligo	26 (54%)
	Multiple	12 (25%)
	n/a	1 (2%)
Chemotherapy	3 months before Radiotherapy	14 (29%)
	Concurrent	9 (19%)
	3 months after radiotherapy	2 (4%)
	3 months before and after Radiotherapy	0
	None	23 (48%)
Immunotherapy	3 months before Radiotherapy	13 (27%)
	Concurrent	2 (4%)
	3 months after radiotherapy	3 (6%)
	3 months before and after Radiotherapy	1 (2%)
	None	29 (60%)
Location of primary diagnosis	Lung	29 (63%)
	Skin	4 (9%)
	Liver	4 (9%)
	Rectal + Colorectal	2 + 1 (7%)
	Kidney	2 (4%)
	Other	4 (9%)

#### Therapy details

As radiotherapy is part of a multidisciplinary therapy, 25 patients received systemic chemotherapy and 19 patients received immunotherapy either 3 months before, concurrent or 3 months after radiotherapy respectively. Median GTV was 21 cm³ and a median PTV margin of 3 mm led to a median PTV size of 37 cm³. 10 patients received 3 fractions with a median prescription dose of 40.5 Gy for 3 and a BED 10 of 95 Gy. 38 patients received 5 fractions with median prescription dose of 40 Gy and a BED10 of 72 Gy. 71% of the fractions were online adapted and 96% patients were treated using DIBH or DEBH. See Table 5 for more details.

**Table 5:** Therapy details

Gross Tumor Volume (GTV) [cm³]	Median (range)	21 (2.2–383)
Planning Target Volume (PTV) [cm³]	Median (range)	37 (5.1–517)
PTV margin expansion [mm]	Median (range)	3 (3–6)
Numbers of Fractions	Median (range)	5 (3–5)
	3 fractions, N (%)	10 (21%)
	5 fractions, N (%)	38 (79%)
BED10 [Gy]	Median (range)	72 (43–112)
3 Fractions		
Prescription Dose [Gy]	Median (range)	40.5 (24–45)
BED10 [Gy]	Median (range)	95 (43–112)
PTVopt Dmax [Gy]	Median (range)	62 (31–75)
5 Fractions		
Prescription Dose [Gy]	Median (range)	40 (30–50)
BED10 [Gy]	Median (range)	72 (48–100)
PTVopt Dmax [Gy]	Median (range)	49 (32–68)
Conformity Index (CI)	Median (range)	0.98 (0.39–1.39)
Homogeneity Index (HI)	Median (range)	1.25 (1.05–1.86)
Number of adapted fractions	Median (range)	4 (0–5)
Respiratory Motion Management Technique		
Deep inspiration breath hold (DIBH)	N (%)	39 (81%)
Deep expiration breath hold (DEPH)	N (%)	7 (15%)
Free breathing	N (%)	2 (4%)

#### Outcome

**Fig. 1:**
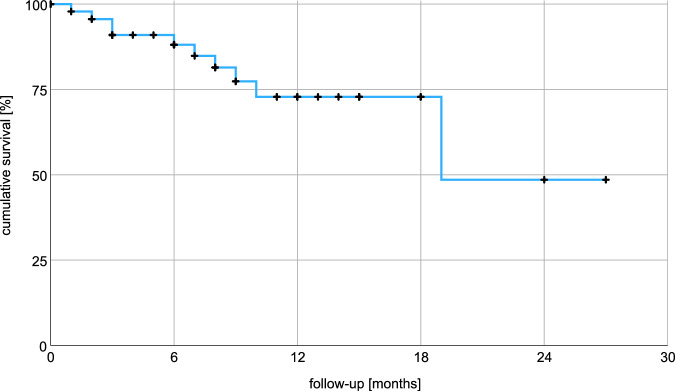
Cumulative survival using Kaplan–Meier analysis. Kaplan-Meier analysis shows a median overall survival of 19 months. Average survival was 19.4 months with 95% confidence interval between 15.2 and 23.6 months.

**Fig. 2:**
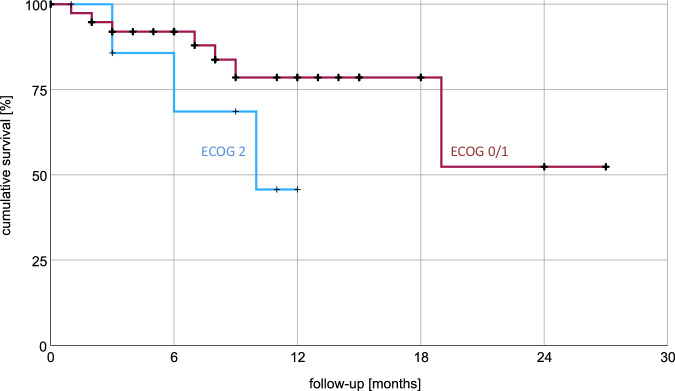
Impact of ECOG status on cumulative survival. Overall survival stratified by ECOG performance score indicated a mean overall survival of 20.4 months for ECOG 0-1 versus 9.2 months for ECOG 2; the median OS for ECOG 0–1 was not reached after 27 months.

**Fig. 3:**
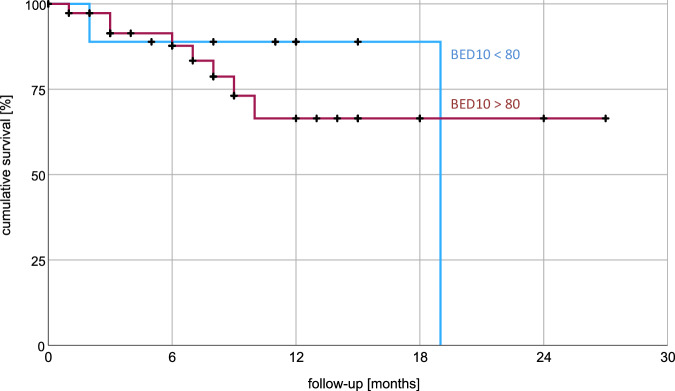
Impact of number of BED10 on cumulative survival. Kaplan–Meier analysis did not identify BED10 as a significant factor influencing overall survival.

Median follow-up time was 8.5 months. Radiotherapy achieved complete or partial remission in 48% of patients and stable disease in 40%. Progressive disease occurred locally in 8% of patients resulting in a median OS 19.0 months (average 19.4, 95% CI: 15.2–23.6) using Kaplan-Meier analysis as seen in Fig. 1. 23% of patients experienced acute toxicity, including 13% with CTCAE grade 1 and 10% with grade 2 toxicities. Fatigue was the predominant toxicity, affecting 17% of patients. Gastrointestinal toxicities were reported by 4% of patients. There were no reports of grade 3 or 4 toxicities. Additional data are available in Table 6. 1-year and 2-year OS were 73% and 48% respectively. Overall survival stratified by ECOG-PS indicated a mean OS of 20.4 months for ECOG 0–1 versus 9.2 months for ECOG 2; the median OS for ECOG 0–1 was not reached after 27 months. The log-rank (Mantel–Cox) test revealed no significant difference in survival distributions with χ² = 1.8 and *p* = 0.18 (Fig. 2). Kaplan–Meier analysis did not identify gender, metastatic pattern, remission status, treatment of the primary tumor, or BED10 (Fig. 3) as significant factors influencing overall survival. BED10 however, was significantly associated with complete remission. Whereas 5 out of 10 patients with BED of 80 Gy or more showed complete remission, only 5 out of 36 patients with BED less than 80 Gy did so, *p* = 0.027 by the two-sided Fisher test.

**Table 6:** Outcome

Median followup [months]	Median (range)	8.5 (0.5 – 26.9)
Acute toxicity	Fatigue	8 (17%)
	Gastrointestinal	2 (4%)
	Anorexia	1 (2%)
	None	35 (73%)
	Not reported	2 (4%)
Local control	Complete remission	10 (21%)
	Partial remission	13 (27%)
	Stable disease	19 (40%)
	Progressive disease	4 (8%)
	n/a	2 (4%)
	Complete remission BED < 80 Gy	5 (14%)
	Complete remission BED > 80 Gy	5 (50%)
OS [months]	Median OS	19.0
	Mean OS (95% CI)	19.4 (15.2–23.6)
ECOG 0-1	Mean OS (95%CI)	20.4 (15.9–24.8)
ECOG 2	Mean OS (95%CI)	9.2 (6.6–11.8)
BED10 < 80 Gy	Mean OS (95%CI)	20.0 (15.6–24.0)
BED10 > 80 Gy	Mean OS (95%CI)	17.3 (12.8–21.8)

#### Power analysis

Overall survival was 20.4 ± 2.2 months for ECOG 0–1 (*n* = 40) and 9.2 ± 1.3 months for ECOG 2 (*n* = 8). In this case, the two-tailed *p*-value is less than 0.0001 which seems highly significant. Accordingly, for a two-sided alpha of 0.05, the statistical power of the sample sizes was close to 1.

On the other hand, overall survival was 20.0 ± 2.2 months for BED10 < 80 Gy (*n* = 37) and 17.3 ± 2.3 months for BED 10 > 80 Gy (*n* = 11). In this case, the 95% confidence intervals well overlap (Table 6). For a two-sided alpha of 0.05, the sample sizes yield a statistical power of 0.89.

Sample sizes were two small to distinguish complete remission for BED 10 < 80 Gy (*n* = 5, 14%) and BED 10 > 80 Gy (*n* = 5, 50%). For a two-sided alpha of 0.05, the resulting power was only 0.10.

## Discussion

Regarding the study protocol, the ethics vote, and the patient information material, in future iterations it would be beneficial to explicitly describe methods of secure computing. This would limit the fashion in which data may be processed, and it would make the (commendable) use of secure computing transparent to ethics counsellors and patients. For the purpose of this experiment, at the time of writing of the study protocol the exact mechanism for data sharing was not known, and hence the study protocol and subsequent documents allowed for more lenient alternatives of data processing.

Regarding the cooperation agreement, it has been argued by a law firm involved in the project that secure multiparty computation would constitute a joint responsibility as provided by GDPR: “Where two or more controllers jointly determine the purpose and means of processing, they shall be joint controllers.” (Art. 26 GDPR) Insofar it would be advisable to explicitly frame the parties as “joint controllers” in future iterations of cooperation agreements that foresee secure multiparty computation. The further provisions of Art. 26 GDPR were adhered to in this project, anyway.

As far as written informed consent is concerned, the novel method does have advantages in terms of privacy and data security, but it was discussed with legal experts that this is not something that the patient should have to rely upon or that should be advertised to the patient. In particular, the new method must not be abused to circumvent any stipulations. In particular, there must not be “less” consent for the novel method to be employed than for conventional open data sharing between the joint controllers. Also, it was recommended by a specialist law firm that the method should be legally considered pseudonymization, rather than anonymization, of the data. For this reason, patients were informed in the consent form that their “personal data and clinical findings will be processed in encrypted (pseudonymized) form […] and will be shared with cooperation partners of the study […] for scientific evaluation.” The cooperation partners abroad were also explicitly named. We are not aware of any challenges obtaining informed consent.

Regarding the evaluation, Rmind^28^ is an exploratory tool that allows fast calculations and provides Statistical Disclosure Control (SDC) on top of Secure Multiparty Computation. In a more regular setting with repeat computations, the SecreC language could be considered as a more stringent alternative. Here, the parties agree on some bytecode that is distributed to all compute nodes and is guaranteed to perform the agreed upon function in a tamper proof way.

Regarding data flow, it would be preferable to keep it entirely server-side from the start. (“Keep people away from data.”) With Federated Secure Computing, data could be sourced server-side. There would be control flow from the researcher to the Federated Secure API, but no data flow outside of the secure server cluster.

As far as interoperability is concerned, it has been discussed to integrate the Federated Secure Computing (FSC) middleware into the infrastructure of the local Data Integration Centers. Data could then be sourced directly from clinical data management systems. Similarly, the FSC middleware could fetch data from Fast Healthcare Interoperability Resources (FHIR). Again, this would be advantageous as data would be processed entirely server-side. While the ability of FSC to source from FHIR server has been tested, it was not employed during this project for lack of preparation of the data as a FHIR.

In general, the implementation of Secure Multiparty Computation (SMPC) is a substantial technical challenge^25^. The complexity of the technology and the required specialist knowledge to implement and operate SMPC solutions has probably hindered its more widespread adoption so far. In this project, we solved the problem in the following way. First, we did not implement our own cryptographic routines or employ an open source SMPC framework. Rather, we licensed an industry-grade SMPC suite. The installation and operation of this suite was not straightforward, but manageable and of similar difficulty as any server-side productivity solution. Secondly, we did not require scientists to operate the SMPC software itself or interact with the server-side. Rather, we provided them with the Federated Secure Computing^25^ middleware. This middleware encapsulates the complex server-side functionality into simple, easy-to-use microservices and exposes them through a web-hosted Application Programming Interface (API). The scientists were then provided with a simple script they had to run locally once to encrypt and upload their data to their server. This removed most of the technical difficulties. The going live of local data was moderated in a video call that lasted about half an hour. Logistical challenges were only encountered in slightly different data formats (especially different locales for calendar formats) and local firewall rules that had to be temporarily adapted during the data upload.

As far as scalability is concerned, we expect that our results will hold in multicenter settings with more than two sites providing data. Basically, every site would need their own FSC server, however FSC is extremely lightweight, and any small virtual machine with a single core would suffice. The much more demanding hardware for SMPC, on the other hand, does not even have to scale at all, as three independent compute nodes are sufficient to provide security to an arbitrary number of data providing parties. This is at least true in our architecture; other solutions might opt for one dedicated compute node per party. Hardware demand would then scale linearly with the number of sites. Data federation times, due to parallel networking, would mainly scale linearly with the size of the data rather than the number of sites. For our small dataset, data upload time was about one second, mainly due to network latency. On the timescales of a clinical study (months to years) we cannot see how data upload would ever be a bottleneck. As regards computing times, the scaling depends on the implementation of the underlying algorithms which are closed source. In most cases, however, we expect computing times to scale linearly or at most quadratically with the number of sites. As the number of sites is naturally limited in multicenter studies, we do not expect the number of sites to be a limiting factor. There is a lot of literature on space and time complexity of SMPC. In practice, multicenter clinical studies would probably not really be limited by SMPC after the initial challenge of setting up the infrastructure.

From a medical standpoint, this novel approach of computation provides significant advantages for healthcare professionals, patients, hospitals, and the broader scientific community. It facilitates the secure and efficient sharing of patient data across multiple centers and countries, all while adhering to stringent data protection rules. This capability allows for the pooling of patient data from different institutions and enables the assembly of larger cohorts, particularly for rare cancers or specialized techniques like MR-guided radiotherapy (MRgRT) or reirradiation. These rare cases often face the challenge of small sample sizes. With the approach presented in this study, valuable data from different regions can be combined. Thus offering insights that would otherwise be impossible to obtain without data sharing.

For hospitals, this results in more efficient research processes and improved resource allocation, as patient data can be collected and analyzed more quickly across diverse populations. For patients, the impact is significant. By enabling the pooling of data from multiple centers, treatments can be better evaluated, and outcomes can be improved through shared insights while still protecting their privacy. This collaborative approach not only speeds up the generation of high-quality clinical evidence but also ensures that patients with rare conditions have access to the latest advances in care. Ultimately, this technique enhances global healthcare by accelerating research, improving treatment options, and fostering international collaboration, all while maintaining the highest standards of data security.

As a pilot study, our analysis focused on the technical aspects with data from two centers and 48 patients. Despite the small sample size, it showed favorable overall survival after radiotherapy. However, the survival advantage for patients with ECOG-PS of 0–1 compared to those with ECOG-PS of 2 was not statistically significant. This observation is consistent with existing literature that suggests comorbidities independently influence survival, irrespective of tumor characteristics or therapeutic interventions. Notably, Wang et al.^29^ identified ECOG-PS as a prognostic indicator for overall survival in a cohort of 600 HNSCC patients undergoing pre-radiochemotherapy, a finding echoed by Kang et al.^30^ who demonstrated the significance of ECOG-PS in predicting overall survival in 714 early-stage non-small cell lung cancer patients treated with stereotactic ablative radiotherapy.

In terms of treatment efficacy, 88% of patients achieved at least stable disease. This outcome aligns with the findings from larger studies, such as those by Chen et al.^31^ who reported pooled 1-year and 2-year local control rates of 84% and 70%, respectively, in a meta-analysis involving 1006 patients with adrenal metastasis. They also reported comparable 1-year and 2-year overall survival rates of 72% and 47%. While Chen et al.^31^ and Ugurluer et al.^32^ proposed a correlation between radiation dose and local control, our analysis did not conclusively demonstrate this relationship significantly, although a higher local control in terms of complete remission was observed in patients with a BED10 of 80 Gy or higher. The analysis did not establish a significant relationship between BED10 and overall survival. The inability to establish a significant relationship between BED10 and overall survival is likely attributable to the small number of patients in our study.

Despite the administration of high doses, toxicity remains low in our study, with only 23% of patients experiencing any side effects and no reports of Grade 3 or 4 toxicities. In contrast, Holy et al.^33^ reported gastric and duodenal ulcers in 2 out of 18 patients following SBRT with a BED10 of 72 delivered to adrenal metastases using conventional C-arm linear accelerators. Despite not exceeding dose constraints for the stomach and duodenum, the authors suggest that variations in organ filling, which were not detectable by X-ray based image guidance, might have contributed to this elevated toxicity. Another study by König et al.^34^ which also treated adrenal metastases with SBRT using conventional C-arm linear accelerators but with daily kilovoltage (KV) or megavoltage (MV) cone-beam computed tomography (CBCT). Treatments were adjusted if there were changes in the filling of the duodenum or stomach, resulting in lower toxicity rates of 32.2% for Grade 1 and 2, with no Grade 3 toxicities reported.

Comparatively, studies like Ugurluer et al.^32^ reported a 9.7% toxicity rate for CTCAE Grades 1-2 using MR-guided SBRT (MRgSBRT), with no Grade 3 toxicities. Our analysis revealed a 23% rate of Grade 1–2 toxicities, primarily fatigue (17%), and also found no Grade 3 toxicities, underscoring the advantages of MRgSBRT over conventional SBRT. The ability of MRgSBRT to adjust daily for anatomical changes and target volumes allows for potentially higher tumor doses while reducing toxicity rates.

The rigor of the study and the generalizability of the outcomes would have been strengthened by a control group. However, the study was explicitly approved by the ethics committee without control group, as it would have been difficult to justify the exclusion of the control group from the tangible benefits of MR guided treatment which is without downside to the patient. In particular, the irradiation scheme and fractionation used in this treatment have only become possible with the introduction of MRgSBRT. Forming a control group would have been difficult as those would have received an entirely different treatment regime. Lastly, a control group would have further reduced the already small sample size. Instead, this study included all patients “ab initio”, i.e. from the introduction of MRgSBRT at the centers.

Further limitations of this study include the restrospective evaluation and the relatively small patient cohort. The latter factor is comparable to other studies given that SBRT for adrenal metastases is not widely available to many patients. Moreover, this study was designed to validate the concept of Federated Secure Computing to enable larger multicenter studies, which would allow for better analysis of these rare diseases while ensuring data protection.

Another limitation is the retrospective analysis of collected data and the development of federation technology during and after data collection. The latter should have a limited effect on the evaluation, as the validity of the evaluation should not be affected by the algorithms used.

This further demonstrates that data federation and research into federation technologies may be helpful to reach meaningful insights in bicentric and multicentric clinical studies for rare tumor entities.

This pilot features secure multiparty computation as a viable and valuable tool in the European data space for evaluation of patient data. Its use has been successfully demonstrated in the setting of a clinical observational study. As far as radiation oncology is concerned, our analysis suggests that MR-guided SBRT may be an effective option for stereotactic radiotherapy of adrenal metastases, offering high local control and low toxicity rates, potentially attributable to the online adaptation workflow.

## Methods

### Clinical study

This research involving human research participants has been performed in accordance with the Declaration of Helsinki, approved by Ethikkommission bei der LMU München, reference number 20–291. Written informed consent was obtained from all participants.

This study aimed to assess the efficacy of MR-guided radiotherapy (MRgRT) in treating adrenal gland metastasis, a context where precision is crucial. Stereotactic body radiation therapy (SBRT) is employed in radiation oncology to deliver high radiation doses directly to tumors, minimizing exposure to surrounding organs and adjacent tissue. Traditional imaging modalities, such as Conebeam CT, often lack the resolution needed for utmost precision, necessitating wider safety margins and consequently, greater radiation exposure to adjacent tissues. The introduction of MR-Linac systems introduces the capability for real-time MRI scanning before and during radiation sessions. This innovation allows for on-the-fly adjustments to account for daily anatomical shifts and the dynamic movement of tumors, especially due to respiratory motions. Such adaptability is essential for treating metastases in the liver or adrenal glands, where tumors are located near critical organs and are prone to move with each breath. MRgRT’s ability to minimize safety margins while concentrating radiation doses on the tumor enhances the precision of treatment, improves local control, and substantially lowers the risk to nearby organs at risk (OARs), marking a significant step forward in the management of complex cases like adrenal gland metastases.

Radiation therapy of adrenal gland metastases is relatively uncommon, largely because they frequently undergo surgical removal. This rarity poses challenges for evaluating and conducting in-depth analyses of radiation treatments, as the number of patients treated at any given center is often insufficient. Furthermore, MR-Linac treatments are not widely available, being offered in only a select number of centers, and they have yet to be established as the standard of care. As a result, many referring physicians might not be familiar with these treatment possibilities, leading to patients receiving radiotherapy elsewhere and not being accounted for in centralized analyses. These factors contribute to the limited representation of such cases in larger studies, underscoring the importance of collaborative data gathering across various institutions and countries to create a comprehensive dataset for meaningful research. Nonetheless, efforts to merge data from different sources are hindered by strict data privacy laws, with Germany being a notable example. Such regulations challenge the sharing of data between healthcare facilities. Secure multiparty computation presents a viable solution by enabling joint data analysis while allowing sites to retain control over their data and protecting patient privacy. Employing this technique improves the possibility of carrying out detailed studies on rare cancers in extensive basket trials, overcoming privacy obstacles and advancing the field of personalized oncology.

During this prospective observational study, the results of MR-guided radiation therapy were documented and evaluated as a measure of quality control (QC). Foremost, this was required for the quality assurance (QA) process in routinely applied radiotherapy at the MR-Linac. All patients that had an indication for MR-guided radiotherapy were treated according to existing standard operating procedures (SOP) and national and international guidelines. The latter were based on clinical data and account for general requirements of the Quantitative Analysis of Normal Tissue Effects in the Clinic (QUANTEC) and American Association of Physicists in Medicine (AAPM) reports. During follow-up care as clinically indicated and legally required, parameters of tumor control and overall survival were routinely documented, as well as toxicities according to Common Terminology Criteria for Adverse Events (CTCAE) v5.0. By the systematical documentation of this data, the long-term effectivity and safety of MR-guided radiation therapy was to be improved for the patients.

This study was designed as a bicenter, prospective, longitudinal observational study without a control group between LMU University Hospital in Munich, Germany, and Fondazione Policlinico Universitario Agostino Gemelli IRCCS in Rome, Italy. There were no changes to the radiooncological treatment plan of any patient. The treatment itself was administered solely per the pre-existing SOPs, based on best available evidence. There was no deviation from standard therapy planned. The documentation of toxicity was part of clinical routine. Clinical results were documented for quality control and quality assurance after the end of treatment and evaluated after the fact.

### Secure multiparty computation

Instead of data being transmitted between the two sites for evaluation in a pseudonymised way, the data was to remain with the two clinical sites and was evaluated by jointly running a secure multiparty computation that will prevent each site from reconstructing the other sites’ data or processing them in a way that was not initially agreed on. Additionally, administrative data protection measures were put in place, and a novel safety architecture and technical infrastructure was implemented.

Administrative data protection measures included an amendment of the original (monocentric) study protocol to allow for multicentric data gathering and joint evaluation; a cooperation agreement between LMU University Hospital, Fondazione Policlinico Universitario Agostino Gemelli IRCCS, and LMU Munich stipulating the joint evaluation of data and the shared responsibility; written informed consent by all patients with details on data storage and processing; an amended institutional ethics vote that provided for joint data processing; a detailed description of the software infrastructure; a data flow diagram; a description of the hardware; a roles and access concept; written signed declarations of the duties of the responsible scientists for systems access; a documentation of technology design and privacy-friendly default settings; a software license agreement; an oral presentation to and deliberation of the institutional Data Use and Access Committee; and a GDPR compliant description and documentation registered by the official Data Protection Officer. In total, the documentation included 98 pages.

**Fig. 4:**
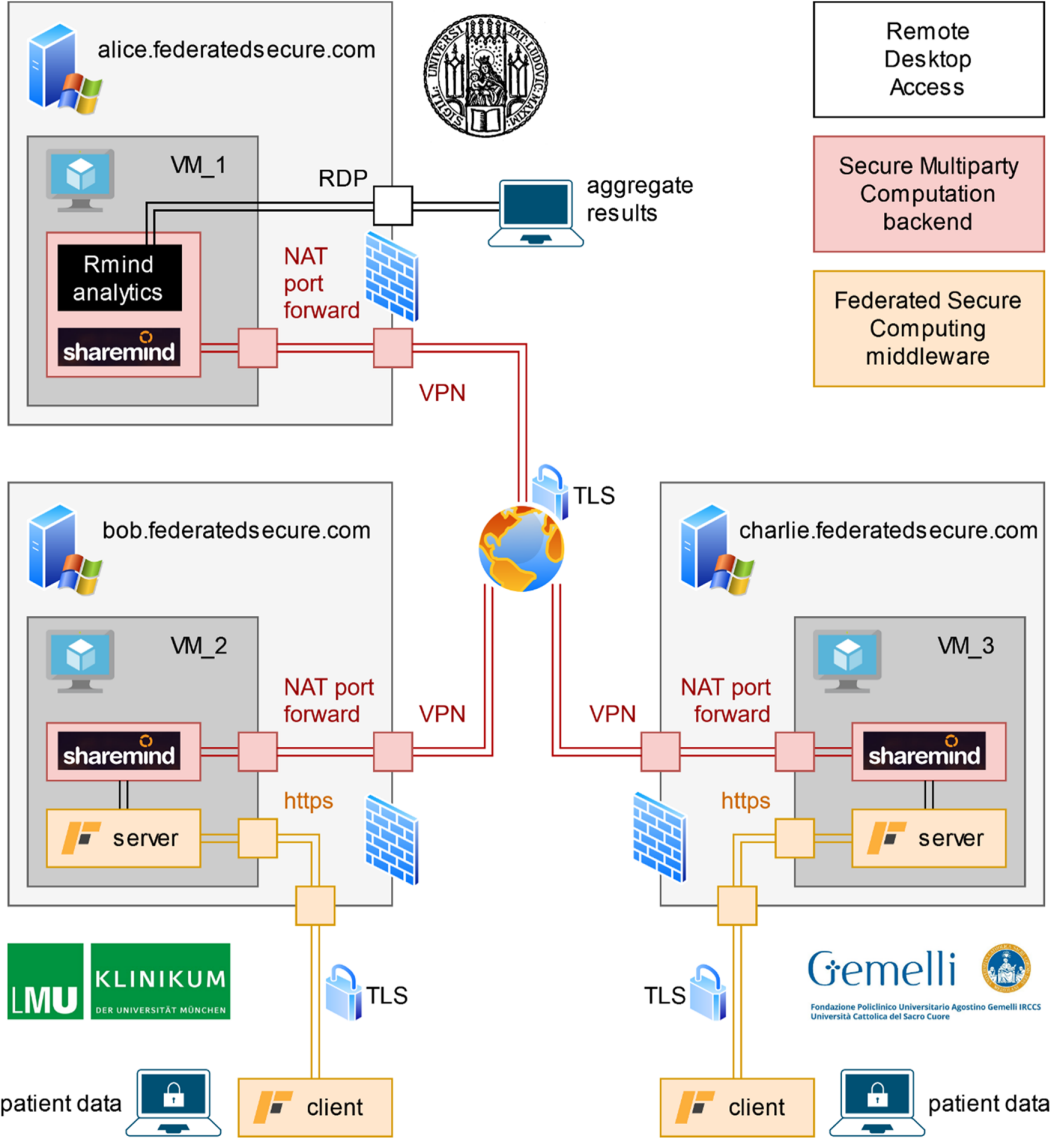
Architecture for secure multiparty computation. Technical data protection measures included a secure multiparty computation backend; a federated secure computing middleware; sandboxing; firewalls and port forwarding; virtual private networks (VPN) and transport layer security (TLS).

Technical data protection measures included a secure multiparty computation backend; a federated secure computing middleware; sandboxing; firewalls and port forwarding; virtual private networks (VPN) and transport layer security (TLS). The resulting architecture is depicted in Fig. 4.

There are three servers connected in a peer-to-peer network secured by VPN and TLS. Their firewalls are configured to only allow ingress from each other and from a researcher by LMU University Hospital, Fondazione Policlinico Universitario Agostino Gemelli IRCCS, and LMU Munich, respectively.

The servers each host an instance of Sharemind MPC, a framework for secure multiparty computation. Data is stored as distributed secret shares across all three servers such that security is guaranteed if there is no collusion between parties. To evaluate data in a privacy-friendly way, the servers of the parties must actively cooperate in all calculations.

The two servers assigned to the two clinical parties in addition host Federated Secure Computing, a middleware that facilitates data input and allows to further restrict data flow to a predetermined protocol. Using Federated Secure Computing as an entry point, the clinical researchers may upload their data which is then immediately converted into secret shares across the three servers. At no point is any data uploaded visible or accessible to any one party.

The third server run by LMU Munich featured an additional instance of Rmind analytics^28^, a secure R-style programming language that offers a suite of statistical functions to be run on secret shares.

In summary, the two clinical researchers had no access to their data vice-versa, and the researcher by LMU Munich had only access to aggregate results but no access to raw data of either clinic.

**Fig. 5:**
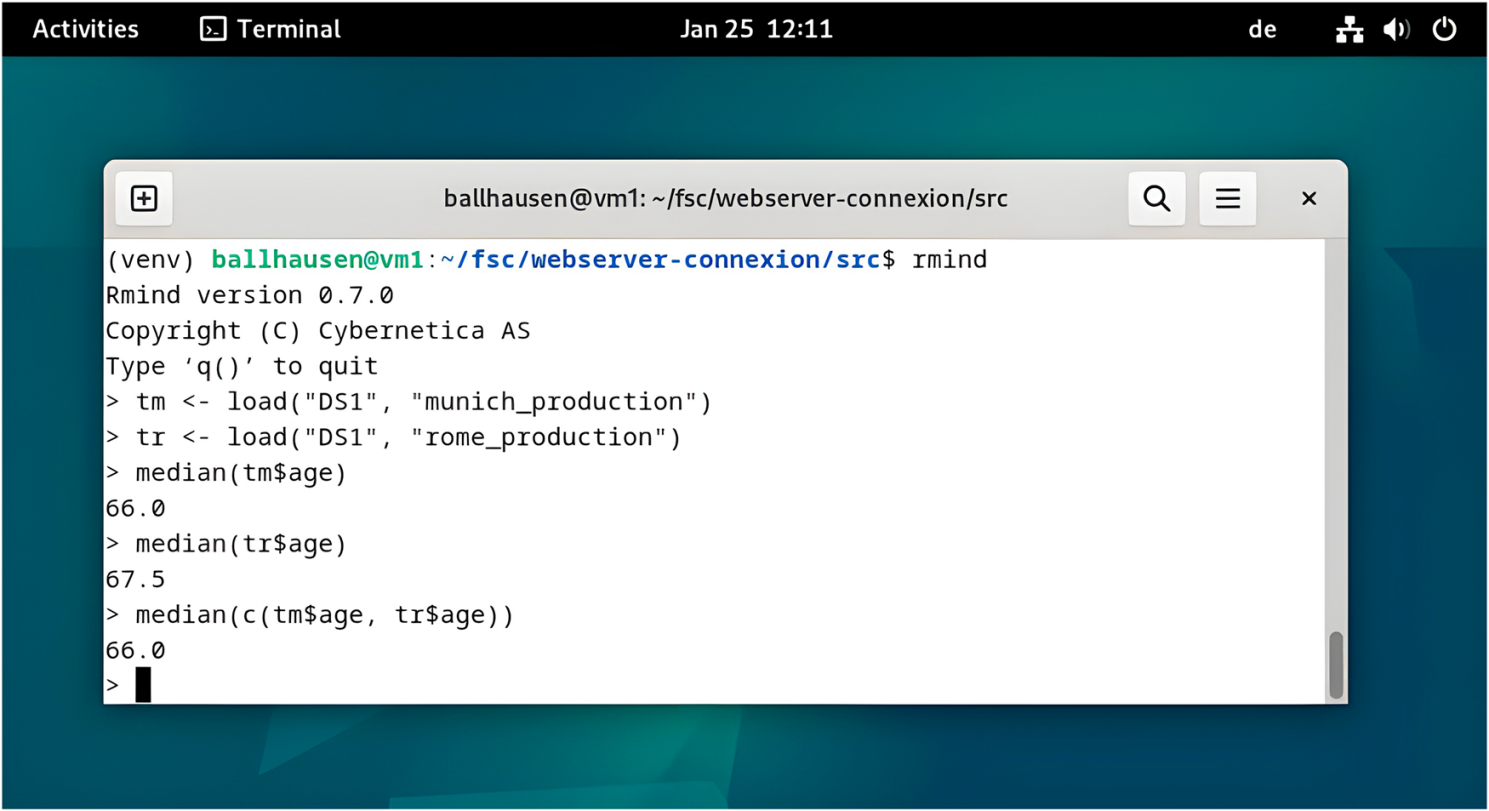
First secure evaluation of patient data from two European partner sites. Commands to the secure computing cluster were given through Rmind. The secure computing cluster performed statistics on the encrypted federated dataset. Only aggregate statistics such as medians are revealed to the researcher, while individual data points remain secret.

Evaluation was performed with Rmind, an interactive statistics environment similar to R offering protection for input and outputs of the study. The Rmind analytics suite is part of Sharemind MPC. Computations in Rmind can be performed through commands given by the researcher, see Fig. 5. Note that Rmind distinguishes between “private” and “public” variables. Private vectors are distributed as secret shares to all Sharemind MPC compute nodes and cannot be directly accessed by the researcher in any way. The researcher may only interface with the cluster as a whole and compute a set of predetermined functions, such as averages, quantiles or frequencies that deliver aggregate results as public variables.

In our case, the input data from Munich and Rome was loaded as private tables “tm” and “tr”:


> tm <- load("DS1", "munich_production")> tr <- load("DS1", "rome_production")


Specific columns such as gender, age etc. were referenced as, e.g.


> tm$age private-float32-vector> tr$gender private-factor-vector


Note that the resulting vectors were private, their values could not be accessed by the researcher directly. They could be used as inputs for aggregate statistics only, e.g.,


> quantile(tm$age) {38.0, 62.5, 66.0, 72.25, 91.0}> freq(tm$gender) [male = 16, female = 8]


Most importantly, the data from Rome (*N* = 24 patients) and Munich (*N* = 24 patients) was concatenated into a larger dataset of *N* = 48 patients:


> length(tm$age) 24> length(tr$age) 24> length(c(tm$age, tr$age)) 48


In this way, quantiles and frequencies of the overall patient population were computed, e.g.,


> freq(c(tm$gender, tr$gender))[male = 29, female = 19]> quantile(c(tm$age, tr$age)){38.0, 60.75, 66.0, 73.0, 91.0}


Finally, subsets for specific events (such as dead/alive during follow up) and subgroups (e.g., by Eastern Cooperative Oncology Group (ECOG) Performance Status) were selected. Survival tables were then constructed by counting events per follow up month, e.g.,


> freq(c(subset(tm, event_death == 0)$follow_up, subset(tr, event_death == 0)$follow_up))[0 = 2, 1 = 1, 2 = 2, …]> freq(c(subset(tm, event_death == 0 & ecog_status == 0)$follow_up, subset(tr, event_death == 0 & ecog_status == 0)$follow_up))[10 = 1, 11 = 0, 12 = 0, …]


The resulting survival tables were then imported into SPSS version 29.0.2.0 for regular survival analysis.

### Administrative data protection measures

#### Study protocol

The original study protocol defined a multicentric, prospective, longitudinal observational study without control group. Treatment was determined by established SOPs according to best available evidence. Clinical results were to be evaluated for quality control in a “pseudonymized” fashion. Electronic processing of patient data was announced. Data protection measures were to be taken. Privacy of individual patients was to be protected and patient data protection was to be implemented. For the purpose of this experiment, the study protocol was then amended to allow sharing of pseudonymized data among the partner sites in a limited fashion.

#### Cooperation agreement

A cooperation agreement was signed by all three partner sites: the two data holding university hospitals and LMU Munich as a neutral third party responsible for evaluation. The cooperation agreement stipulated that “Each Party covers its own risk, assumes responsibility for its own actions and omissions and shall insofar hold harmless – the other Parties against claims brought by third parties” and that “Each Party commits to comply, within its own sphere, with applicable legislation on personal data protection”.

#### Patient information

Written informed consent stated the usual terms (why is the study conducted, how is the study conducted and what are the risks involved, voluntary enrollment and exit). In terms of data protection, the written informed consent contained the following information:

- Lawfulness of processing: Consent by the subject for specific purposes. (Art 6 GDPR 1. a).
- Responsible entity for processing: University hospital, represented by CEO / Chief Medical Officer and Commercial Director and Official Data Protection Officer with contact details.
- Medical confidentiality: All legal requirements. In particular, pseudonymized data would be shared with the cooperation partners. Rules for pseudonymization. Minimum required (10 years) and maximum allowed (20 years) retention period.
- Risk of data processing: General information on the risks of data processing and storage. Provision to share data outside of the EU Data Space only to a specific cooperation partner in Switzerland (not used in this study). Sufficient data protection standards in Switzerland.
- Retraction of consent possible at any time. Rights to have data retracted and deleted. Contact person for data retraction and deletion, the Official Data Protection Officer with contact details.
- Contract address for complaints, the Data Protection Officer of the State with contact details.
- Patient signature.

#### Ethics vote

The original ethics vote stipulated the responsibilities for lawful conduct of the study and referred to GDPR. The amended ethics vote consented to data sharing and joint data processing between the cooperation partners: “As part of the clinical study, pseudonymized patient data will be shared in accordance with all relevant data protection standards and legal requirements with [the cooperation partners] for joint processing.”

#### Software description

A 25-page technical description, published in a peer-reviewed journal, was provided to the Data Protection Officers to demonstrate technical data protection measures of the middleware. A 27-page technical description, provided by the manufacturer, was provided to the Data Protection Officers to demonstrate technical data protection measures of the backend.

#### Data flow diagram

A data flow diagram (similar to Fig. 4) with details such as internal server names, static IP addresses and networking details (not included in Fig. 4) were provided to the Data Protection Officers. For each data storage and each data connection, details on the nature of the data were given (individual patient data, aggregate results, control flow).

#### Declaration by personnel, training

University hospital personnel and university personnel had all signed data protection requirements and responsibilities when entering their employment contracts. Personnel by the university hospital was required to regularly refresh data protection knowledge through institutional online training. For the purpose of this study, the external cooperation partner had to individually sign a 7-page “Undertaking for the dealing with personal or otherwise confidential data”. It stipulated rules for lawfulness, fairness, transparency, purpose limitation, data minimization, accuracy, storage limitation, integrity and confidentiality (Art. 5 GDPR) and rules for conduct with confidential data.

#### Description of hardware and backup

A 3-page document listed all hardware and software components with their suppliers and physical location. It stipulated rules for backup; anti-virus precautions; emergency, restart and recovery procedures; technical safety measures; physical safety measures; access control both logical and physical.

#### Technology design and privacy-friendly defaults

A 2-page document recurred to the detailed software description and briefly listed state of the art; implementation cost; pseudonymization; data minimization; privacy friendly default settings.

#### Roles and access concept

A roles and access concept defined three roles:

- Administrator (physical access to servers, configures operating systems, installs applications and packages, responsible for privacy-friendly defaults, responsible for technical data protection such as firewalls and anti-virus)
- Principal Investigator (supervises the participation of the cooperation partners in the joint evaluation; communicates the result of the computation to the cooperation partners)
- Researcher (interacts with the Sharemind MPC backend, interacts with the Federated Secure Computing middleware, has access to the input data of their respective partner site for purposes of quality control and good scientific practice, inputs the data of their respective partner site to the compute cluster)

The Role and Access Concept then individually named persons for each of the roles from the different partner sites and listed the respective means of Access Control (usernames, passwords, temporary one-time keypads).

#### Software license agreement

For each of the three parties, the “Sharemind MPC Customer Agreement” listed exactly one person by name, who were licensed to interact with the software: one principal investigator (role: researcher) from LMU, and each one scientist (role: data owner) from LMU University Hospital and Gemelli IRCCS. The list of these persons conformed to the Roles and Access Concept as above.

#### Data use and ethics committee

The study and the data protection measures were discussed in a regular meeting of the Data Use and Access Committee (DUAC) of LMU University Hospital. The principal investigator of the study is a member of the Data Use and Access Committee and presented the intended project in person. To avoid a conflict of interest, the principal investigator did not take part in the vote of the Data Use and Access Committee. The principal investigator did receive the recommendation of the Data Use and Access Committee and commented on them.

#### GDPR compliant description

A GDPR compliant description containing a structured questionnaire and points 2.1 through 2.11 as above as attachments was provided to the Official Data Protection Officers.

### Technical data protection measures

#### Secure multiparty computation backend

For the experiment, a secure computing network was set up, see Fig. 4. Three identical physical servers (Intel Xeon Silver 4310, 64 GB) were set up as secure computing nodes in a Sharemind MPC network. Two of the three servers simultaneously acted as data input nodes for the two data holding parties, one used by Munich, and one used by Rome.

The data input nodes would protect incoming patient data by secret sharing it and distribute the shares to the Sharemind MPC cluster. They would then wipe any unencrypted in-memory patient data. From this point on, patient data exists only as distributed secret shares and cannot be accessed by either data holding party or even a system administrator of any one compute node. Only by jointly executing previously agreed upon analytics functions, the Sharemind MPC cluster is mathematically able to generate aggregate results (e.g., medians, frequency tables). Access to or evaluation of individual patient data is not possible by design and by intent.

#### Federated secure computing middleware

To facilitate the uploading of patient data, the Federated Secure Computing middleware (FSC) was employed. FSC acts as a layer between the Sharemind MPC backend and the data owning parties. It encapsulates the Sharemind MPC interface and provides only the strictly necessary functionality as microservices through an Open API 3.0 compliant web interface. This allows to separate the concerns of cryptography (server-side) and of medical data science (client-side) and to enforce strict data flow protocols and to restrict control flow. In this case, the only server-side functionality exposed was a CSV import function. Client-side, a small Python script would read patient data from the clinical database, convert it to an agreed upon spreadsheet format and feed the data to the FSC interface. This script was provided to both data holding parties and had to be run by hand once.

#### Sandboxing

The servers ran Microsoft Windows Server 2022 Standard, Version 10.0 Build 20348 as host operating system. Virtualization was provided by Oracle Virtual Box 7.0.0 r 153978. Besides the virtualization software, no other software was run on the host operating system.

The guest operating system was Debian GNU/Linux 12 (Bookworm) with Linux 6.1.0-13-amd64 kernel. The guest operating system was allocated 8 virtual processors (4 cores) and 16 GB of RAM. Besides the Federated Secure Computing middleware and the Sharemind backend, no other software was run on the guest operating system.

#### Firewall and port forwarding

Two special inbound rules were added to Windows Defender Firewall with Advanced Security on the host operating system allowing ingress on TCP port :55000 (Federated Secure Computing) and TCP port :30000 (Sharemind).

Port forwarding between the host and guest operating system was provided in the form of Network Address Translation (NAT) and port forwarding of ports :55000 and :30000. No other ports were forwarded.

#### Virtual private network

Between the three compute nodes, a virtual private network (VPN) was configured through Sharemind MPC settings. The three nodes shared a common namespace “FSCRomeMunich” and identified each other as “FSCVM1” through “FSCVM3”. The VPN was hardcoded through static IP addresses in each compute nodes server settings file, and the three compute nodes would be listening only to ingress from each other.

#### Transport level security (TLS)

The Federated Secure Computation servers enforced TLS for all incoming client connections. ISO/IEC 9594-8 compliant self-signed certificates were generated according to the X.509 ITU-U standard using 4096 bit RSA encryption. In compliance with TLS 1.3, key exchange through the Diffie Helman protocol was enforced for every new connection, providing forward security on the transport layer.

The Sharemind server cluster was similarly using TLS to protect server-server communication. As per Sharemind documentation, 2048 bit RSA public-private key pairs were generated and converted to DER format. The generated public keys were then manually distributed across all Sharemind compute nodes.

#### Software activation

Software activation happened through 20-character key files that were transferred in part by email (12 characters) and in part by Short Message Service (8 characters). The activation keys were then manually installed on each Sharemind compute node.

## Data Availability

Patient data is protected by strict privacy laws and is not generally available to the public. Requests for data sharing may be made through the local data use and access (DUAC) committees.

## Code availability

Federated Secure Computing is available as free and open source software under the MIT license at https://github.com/federatedsecure. Sharemind MPC is available as commercial software from Cybernetica AS, Estonia.
